# 3.5KJPNv2: an allele frequency panel of 3552 Japanese individuals including the X chromosome

**DOI:** 10.1038/s41439-019-0059-5

**Published:** 2019-06-18

**Authors:** Shu Tadaka, Fumiki Katsuoka, Masao Ueki, Kaname Kojima, Satoshi Makino, Sakae Saito, Akihito Otsuki, Chinatsu Gocho, Mika Sakurai-Yageta, Inaho Danjoh, Ikuko N. Motoike, Yumi Yamaguchi-Kabata, Matsuyuki Shirota, Seizo Koshiba, Masao Nagasaki, Naoko Minegishi, Atsushi Hozawa, Shinichi Kuriyama, Atsushi Shimizu, Jun Yasuda, Nobuo Fuse, the Tohoku Medical Megabank Project Study Group, Gen Tamiya, Masayuki Yamamoto, Kengo Kinoshita

**Affiliations:** 1 Tohoku Medical Megabank Organization, Tohoku University, Sendai, Japan; 2 Graduate School of Medicine, Tohoku University, Sendai, Japan; 3 Statistical Genetics Team, RIKEN Center for Advanced Intelligence Project, Tokyo, Japan; 4 Graduate School of Information Sciences, Tohoku University, Sendai, Japan; 5 Advanced Research Center for Innovations in Next-Generation Medicine, Tohoku University, Sendai, Japan; 6 International Research Institute of Disaster Science, Tohoku University, Sendai, Japan; 7 Iwate Tohoku Medical Megabank Organization, Iwate Medical University, Morioka, Japan; 8 Miyagi Cancer Center, Miyagi Hospital Organization, Natori, Japan; 9 Institute of Development, Aging and Cancer, Tohoku University, Sendai, Japan

**Keywords:** Rare variants, Structural variation

## Abstract

The first step towards realizing personalized healthcare is to catalog the genetic variations in a population. Since the dissemination of individual-level genomic information is strictly controlled, it will be useful to construct population-level allele frequency panels with easy-to-use interfaces. In the Tohoku Medical Megabank Project, we sequenced nearly 4000 individuals from a Japanese population and constructed an allele frequency panel of 3552 individuals after removing related samples. The panel is called the 3.5KJPNv2. It was constructed by using a standard pipeline including the 1KGP and gnomAD algorithms to reduce technical biases and to allow comparisons to other populations. Our database is the first large-scale panel providing the frequencies of variants present on the X chromosome and on the mitochondria in the Japanese population. All the data are available on our original database at https://jmorp.megabank.tohoku.ac.jp.

## Population genetics: large database of Japanese gene variations constructed

A new database provides information on the frequency of genetic variations within 3552 Japanese individuals, and facilitates comparisons with other populations. The reference panel, constructed by Kengo Kinoshita of Tohoku University, Sendai, and colleagues in Japan is also the first large-scale database to provide genetic variation frequency information on the X chromosome and mitochondrial DNA in the Japanese population. The methods used to sequence the genetic data are similar to those used in other large databases, allowing comparisons with other populations. The population size and methods used to compile the database overcome limitations in previous Japanese reference panels. This and similar databases that catalog genetic variations within populations can improve efforts towards personalizing healthcare and contribute to the study of human population genetics. The database is publicly available online.

## Introduction

It is of fundamental importance to catalog the genetic variation in a general population to realize personalized healthcare and personalized medicine. Since different populations show divergent genetic variations, population-specific analyses based on large cohorts are required^1,2^. Since individual-level genomic information is classified as personal data, access to it is strictly controlled. Therefore, allele frequencies have been published in the form of reference panels^3,4^ to clarify population-level differences.

Accordingly, a large allele frequency reference panel based on the genomes of 1070 Japanese individuals was first published in 2014^5,6^ by the Tohoku Medical Megabank (TMM) Project^7^. A subsequent version was published in 2016 that included 2049 individuals^8^, and one distributed in 2017 included 3554 individuals. These reference panels were used for various other projects. For example, the IRUD project (Japan’s Initiative on Rare and Undiagnosed Diseases^9^) used the reference panel to reduce the discovery of false positive single nucleotide variants (SNVs) during the exome analyses of undiagnosed patients. In another project, CYP SNVs included in the reference panels were selected and analyzed systematically for their effect on drug metabolism^10–12^. As seen from these examples, the previous versions of the reference panels worked well; however, there are some limitations. One of the limitations was the lack of multiallelic sites as predicted by the infinite site model^13^. Following standard practice, multiallelic sites were removed from the previous frequency panels, which resulted in a lack of high frequency alleles in the reference panel. Since human genomes are now considered to have accrued a large number of mutations due to a rapid expansion of the population size, the analysis of multiallelic sites should prove interesting from the perspective of human population genetics. However, we hope that this will be described elsewhere. Another limitation of our analysis was the gradual obsolescence of the 1KJPN pipeline. When we constructed 1KJPN, several analysis pipelines were used, but now large-scale analyses, such as the 1000 Genomes Project (1KGP)^14^ and the genome Aggregation Database (gnomAD)^15^, use virtually equivalent protocols for variant calling. The difference in pipelines can make it difficult to compare the allele frequencies of different populations. Thus, we decided to perform a reanalysis of variant calls and construct a new reference panel for the Japanese population. In this paper, we will describe some details of our new panel construction by using a pipeline similar to the 1KGP and gnomAD pipelines. We also report the variant frequencies of the X chromosome and those of mitochondria, which makes this the first such report to do so on a large scale for the Japanese population.

## Materials and methods

### Sample information

Data were obtained from 3552 individuals in Japan [Supplementary Table 1]. Among them, 3342 samples came from individuals who participated in the TMM Project, which was led by the Tohoku Medical Megabank Organization (ToMMo) at Tohoku University and Iwate Tohoku Medical Megabank Organization (IMM) at Iwate Medical University. The TMM project recruited participants from both the Miyagi and Iwate prefectures. Individuals who presumably originated from other prefectures were also included [Supplementary Table 1a]. A further 29 samples came from individuals who participated in the Nagahama Study^16^. Finally, 181 samples came from individuals recruited by the National Hospital Organization Nagasaki Medical Center. Written informed consent was obtained from all the participants.

Participants’ declared age and their sex as determined from X and Y chromosome sequencing are presented in Supplementary Table 1b. Samples with irregular karyotypes, such as those with Turner syndrome, were excluded. Close relatives of individual subjects, based on mean identity-by-descent (IBD; PIHAT in PLINK version 1.07) values indicating relatedness closer than between third-degree relatives, were excluded.

### Whole-genome sequencing

Library preparation and sequencing were performed as described earlier with minor modifications^5^. Briefly, genomic DNA extracted from the buffy coat was fragmented by sonication to an average target size of 550 bp. After library quantification using the quantitative MiSeq method^17^, sequencing was performed on the HiSeq 2500 system (Illumina). The TruSeq Rapid PE Cluster V1 and SBS Kits (1 sample per flowcell) and the TruSeq Rapid PE Cluster Kit V2 and SBS Kit (2 samples per flowcell) were used for 162-bp paired-end (162PE) and 259-bp paired-end (259PE) protocols, respectively.

### Whole-genome re-sequencing workflow

We employed a workflow known as the GATK Best Practices workflow, which is becoming the standard procedure globally for whole-genome re-sequencing analysis. Several recent large-scale genome analyses, such as the 1000 Genomes project^14^ and gnomAD^15^, adopted the same workflow. Although we used an original re-sequencing workflow for 1KJPN^5^, 2KJPN, and 3.5KJPNv1, we decided to use a more common pipeline to build 3.5KJPNv2 to allow for comparisons of allele frequencies between different populations. We customized three steps in the GATK Best Practices workflow: (1) the choice of the reference genome, (2) the use of base quality score recalibration (BQSR), and (3) the joint genotyping step.

### Alignment of sequence reads to the reference genome

The FASTQ files of each sample were aligned to a set derived from the human reference genome (GRCh37) that contains the revised Cambridge Reference Sequence (rCRS), unlocalized/unplaced contigs, human gammaherpesvirus 4 sequence (NC_007605), and a decoy sequence (hs37d5). Two pseudoautosomal regions (PAR1 and PAR2) on the Y chromosome are masked as N. This reference genome sequence is referred to as hs37d5.fa and is the same reference sequence as that used in the 1000 Genomes project Phase 2^14^.

FASTQ files were aligned with hs37d5.fa using BWA-MEM^18^ version 0.7.12 and sorted by their coordinates using the SortSam program included in Picard^19^ version 2.10.6. BWA-MEM was run at “-K 10000000”, in addition to the default options to reduce any differences when we performed calculations with multiple threads. Thereafter, duplicate PCR reads were removed by using the MarkDuplicates command in Picard. The output was written into a BAM (Binary Alignment/Map) format file. Such files will be referred to as the baseline BAM files in this study.

Although the GATK Best Practices workflow recommends that the BQSR step be carried out after the mapping, we did not do so for the following reasons. Before analyzing our full dataset of 3,552 samples, we evaluated the effect of BQSR on our dataset. For this purpose, we randomly selected 100 samples from our dataset and re-sequenced them using BQSR as described in GATK Best Practices. We also performed re-sequencing without the BQSR step. Finally, we performed SNP array analyses on both sets of 100 samples. In other words, we checked concordance among two kinds of genotyping results: (i) genotyping results obtained after the incorporation of BQSR and (ii) results obtained without BQSR.

### Variant discovery on autosomes and joint genotyping

Variant calls for each baseline BAM file were made by using the HaplotypeCaller program included in GATK version 3.7, resulting in the generation of genomic VCF (GVCF) files. These were used to perform multisample joint genotyping in the following steps. After generation of GVCFs for all samples, joint genotyping was performed using the GenotypeGVCFs program included GATK version 3.7. Joint genotyping of large samples usually takes a large amount of computational time, but it was not feasible for us to perform joint genotyping of 3500 samples at the same time. To overcome this difficulty, we divided the autosomes into 3 Mb chunks and performed joint genotyping of each chunk across all the samples. After all the chunks were processed, they were concatenated to produce the whole autosome. To avoid edge effects that may be introduced by chunk splitting, we made overlaps of 1 kb between adjacent chunks and checked the concordance of variant calls in the overlapping regions. If discordant variant calls were found in the overlapping regions, we removed them. In total, we found 470 discordant variants on the autosomes, and they were not included in the results.

After merging the chunks and checking their concordance, we applied the Variant Quality Score Recalibration (VQSR) filter. The GATK resource bundle was used as known site information for the VQSR step. We opted for SNV filtration based on the following VQSR scores: QD (variant call confidence normalized by depth of sample reads supporting a variant), MQ (root-mean-square value of the mapping quality of reads across all samples), MQRankSum (rank-sum test for mapping qualities of REF versus ALT reads), ReadPosRankSum (rank-sum Test for relative positioning of REF versus ALT alleles within reads), FS (strand bias estimated using Fisher’s exact test), SOR (strand bias estimated using the symmetric odds ratio test), DP (total depth of coverage per sample and over all samples), and InbreedingCoeff (likelihood-based test for the inbreeding among samples). For INDEL filtration, we excluded the MQ and MQRankSum scores from the preceding list. Finally, we collected the SNVs and INDELs that passed the VQSR step. The numbers of SNVs and INDELs found on the autosomes and the X chromosome are shown in Table 1a.

**Table 1:** Statistics of variants discovered and comparison of WGS genotyping and SNP array genotyping. (a) Number of variants found on autosomes, X chromosome, and mitochondria

			X chromosome		X chromosome		
	Autosomes		(PAR1, PAR2, XTR)		(PAR1, PAR2)		Mitochondria
	Raw	Passed	Raw	Passed	Raw	Passed	Raw
SNVs	51,168,347	44,107,909	2,065,505	1,750,054	2,005,093	1,726,127	2483
INDELs	7,283,992	5,839,667	295,681	240,016	305,477	244,260	–
Multi allelic SNV sites	1,409,934	701,047	48,408	20,139	54,867	28,620	–

**Table 1b:** Comparison of the WGS genotyping procedure (including the BQSR step) and the SNP array genotyping procedure. Numbers in cells correspond to the numbers of SNVs classified by array genotyping and WGS genotyping. The label “Not observed” in the table means that a variant was not observed by either SNP array or WGS

		Array genotype				
		Not observed	No call	0/0	0/1	1/1
WGS (with BQSR) genotype	Not observed	–	0	236	4	1
	No call	9581	0	3	0	0
	0/0	556282	42	16301	28	3
	0/1	119717	33	20	8905	31
	1/1	95637	15	2	11	5339

**Table 1c:** Comparison of WGS genotyping procedure (excluding the BQSR step) and SNP array genotype

		Array genotype				
		Not observed	No call	0/0	0/1	1/1
WGS (with BQSR) genotype	Not observed	–	0	236	4	1
	No call	9791	0	3	0	0
	0/0	552854	42	16301	27	3
	0/1	119012	33	20	8906	31
	1/1	95483	15	1	10	5338

### Variant discovery on X chromosome

**Fig. 1:**
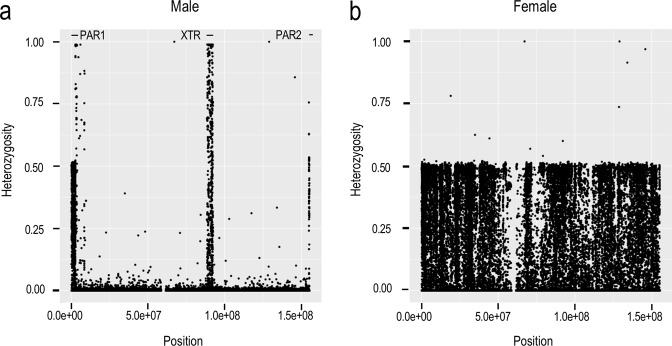
Heterozygosity of the X chromosome observed by SNP array analysis. The three regions showing high heterozygosity in (**a**) are designated par1, XTR, and par2. To perform the variant calls, we used the following regions of GRCh37 corresponding to these three regions: 60,001-2,699,520 (PAR1), 88,456,802-92,375,509 (XTR), and 154,931,044-155,260,560 (PAR2)

The difference between the analyses of the autosomes and those of the sex chromosomes was the ploidy settings for calling single-sample variants during the GVCF file generation stage. It is well known that there are pseudoautosomal regions (PAR) on the X and Y chromosomes that have similar sequences. Thus, variant calling for these regions should be performed with different ploidy settings according to the sex of each sample. The sex of each sample was determined using PLINK (1.9b05). Since the female samples have two X chromosomes, we treated their reads as having originated from the diploid genome and performed variant calling for them just as we did for the autosomes. For the male samples that have one X chromosome and one Y chromosome, we performed variant calling for PAR and non-PAR reads using different ploidy settings. We treated the PAR reads as having originated from the diploid genome, and non-PAR reads as having originated from the haploid genome. The existence of at least two PARs, called PAR1 and PAR2, has been accepted by most international genome projects. However, there is some discussion about the existence of another similar pseudoautosomal region called the X-transposed region (XTR)^20–22^. To check the pseudoautosomal nature of XTR, we observed heterozygosity of the X chromosome for male samples by SNP array analyses. We found significantly higher heterozygosity in three regions, including the XTR, in the Japanese population (Fig. 1). Therefore, we decided to use two different SNV calling procedures: one where only PAR1 and PAR2 were considered and the other where XTR was also treated as a pseudoautosomal region. After the genotyping step, we extracted the unmapped reads and the reads that were mapped onto the X and Y chromosomes from the baseline BAM files and then remapped them onto a modified version of hs37d5.fa in which XTR along with two pseudoautosomal regions on Y chromosome were masked as N. Except for the mapping step, we employed the same variant calling workflow as we did for the autosomes and X chromosome and considered both PAR1 and PAR2.

We constructed X chromosome allele frequency panels for the two ploidy settings by generating the GVCF files and by joint genotyping as described before. We also used the GATK resource bundle as known site information for the VQSR step.

### Variant discovery on mitochondrial DNA

Since the mitochondrial DNA is circular, we analyzed data originating from the mitochondrial genomes by converting the circular DNA sequence into two linear DNA sequences by inserting a breakpoint within it. One of the linear DNA sequences was the same as that used by rCRS, while the other was generated by shifting the breakpoint by 10,000 bases. The shift was introduced to avoid any edge effects at the breakpoint on the variant calls. First, we extracted unmapped reads and reads aligned onto the mitochondrial genome from the baseline BAM files. Thereafter, we realigned them onto the two linear mitochondrial genomes using BWA-MEM version 0.7.12. Afterwards, we used GATK HaplotypeCaller version 3.7 to detect the variants. Mitochondrial genomes are known to be heteroplasmic. However, we ignored this consideration while building the current version of the variant panel because our focus was on determining the major variants in the first step of analyses for the Japanese population. Therefore, we treated the mitochondrial DNA as haploid when we performed the variant calls.

### Variant annotation

Variants found from 3,552 individuals were annotated by snpEff^20^ version 4.3t while using GENCODE^21^ release 28. GENCODE release 28 is not provided as a prebuilt database for snpEff. Therefore, we manually converted the GTF file downloaded from the GENCODE website to a snpEff database according to the instructions given in the snpEff online manual. In addition to GENCODE gene annotations, rs numbers of each variant were resolved by SnpSift^22^ version 4.3t while using dbSNP^23^ release 150.

### Calculation of genome accessibility metrics from BAM files

From the baseline BAM files of each sample, we collected mean depth metrics for each base. We refer to the dataset as the “Genome Accessibility” dataset. Mean depth metrics helps us to identify genome regions where alignment of short reads could not be performed well, mainly due to complexities in the genome sequences. The genome accessibility dataset could be used for two main cases: (i) filtering of variants by depth information and (ii) supporting evidence of the absence of variants in regions when no variants are discovered in the region.

For this purpose, we calculated the mean depth from 500 samples (162PE protocol) and 445 samples (259PE protocol) for each base. To clarify regions where alignment of short reads could not be performed, we calculated mean depth by using all reads aligned to reference genome regardless of mapping quality (MAPQ) of reads and by using the reads with MAPQ >= 20. During the alignment step, sequence reads mapped onto multiple regions on the reference genome will be assigned a low MAPQ value (typically 0), and the difference in mean depth values obtained by the two calculations will reflect whether multimaps of reads are likely to occur. SAMtools^24^ 1.6 was used to retrieve depth information from each BAM file, and an in-house Python script was used to obtain final mean metrics.

## Results and discussions

### Statistics and quality evaluation of variants

We performed whole-genome re-sequencing analyses as described in the Methods section and collected the SNVs and INDELs that passed the VQSR step. The numbers of SNVs and INDELs found on the autosomes and the X chromosome are summarized in Table 1a.

To evaluate the quality of these variants along with the effect of the BQSR step on the final whole-genome re-sequencing workflow, we performed SNP array analyses and observed genotype concordance between WGS and SNP array analyses determined by Japonica Array version 1^25^ for 1036 samples. As a result, we did not observe significant differences in concordance between genotyping results obtained after the incorporation of BQSR and results obtained without BQSR, according to the markers present in the SNP array (Table 2b, c). In light of the preceding information and given that BQSR requires approximately 10 h per sample to execute, increasing the total computation time by 50%, we decided to skip the BQSR step in this study.

**Table 2:** Overview of outliers found in allele frequency comparison plots

	Position	Ref/Alt	3.5KJPNv2	gnomAD EAS	Possible reason
Fig. 2a–(i)	3259463	G/T	0.6337	1.0000	Unknown
	25452223	T/C	0.3705	0.8483	Low complexity region
	35283958	T/C	0.6488	1.0000	Low complexity region
	88051052	T/C	0.5943	1.0000	Unknown
	166721424	C/T	0.4401	0.8750	Unknown
Fig. 2a–(ii)	32609253	G/A	0.5351	0.0777	HLA region (HLA-DQA1)
	(15 SNVs omitted)				
	32629146	G/A	0.5370	0.0037	
Fig. 2a–(iii)	32609379	C/T	0.7194	0.2405	HLA region (HLA-DQB1)
	32610825	A/G	0.7245	0.2357	
	32629257	T/A	0.7793	0.2308	
	32629161	A/G	0.7201	0.0683	
	32629193	C/T	0.7211	0.0293	
	32629247	A/C	0.7171	0.0157	
	Position	Ref/Alt	3.5KJPNv2	RIKEN	Possible reason
Fig. 2b–(iv)	93743452	A/T	0.3535	0.1842	Unknown (gnomAD EAS = 0.3532)

(a) Summary of outliers in Fig. 3(a). The “Position” column shows the chromosomal position of a variant, the “Ref/Alt” column gives the reference allele and the alternative allele, the “3.5KJPNv2” column gives the allele frequency observed in 3.5KJPNv2, and the “gnomAD EAS” column gives the allele frequency observed in gnomAD EAS

(b) Summary of outliers found in Fig. 3b

For the other samples, we also performed concordance analyses to check the quality of genotyping either by Japonica Array version 1 (918 samples), by Japonica Array version 2 (420 samples), by Illumina Omini2.5 (3399 samples) or by Illumina Omni Express Exome (408 samples). In short, all of the samples included in 3.5KJPNv2 had both whole-genome sequence data and genotyping results by some SNP arrays. We observed that most samples have a high concordance (>=99.0%). Supplementary Fig. 1 shows a histogram of the concordance ratio of the re-sequencing result and Illumina Omni2.5 SNP array genotype.

### Comparison with other reference panels

**Fig. 2:**
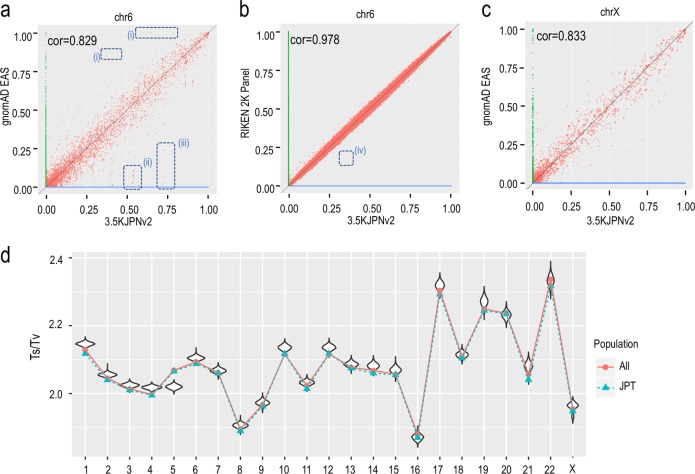
Comparisons of 3.5KJPNv2 with other genome data. **a** Comparison of allele frequencies of variants on chr6 between gnomAD EAS and 3.5KJPNv2. Red dots represent alternative allele frequencies in each population (x-axis: 3.5KJPNv2, y-axis: gnomAD EAS). The green and blue dots show SNVs lacking either in 3.5KJPNv2 or genomAD EAS, respectively. Some outliers denoted by broken circles are described in the text. **b** Comparison of allele frequencies of variants on chr6 between the RIKEN 2 K panel and 3.5KJPNv2. Colors are used in the same way as in (**a**). **c** Comparison of allele frequencies of variants on the X chromosome between gnomAD EAS and 3.5KJPNv2. Colors are used in the same way as in (**a**). **d** Distribution of Ts/Tv values for each chromosome. Violin plots were generated from the 3.5KJPNv2 data. The red and green lines are the average values of all 1KGP samples and 1KGP-JPT samples, respectively. In the calculation of Ts/Tv of variants on the X chromosome, only female samples are used. (1KGP ALL: 1271 samples, 1KGP JPT: 48 samples, 3.5KJPNv2: 1999 samples)

We compared the allele frequencies obtained by us as part of 3.5KJPNv2 to those of the whole East Asian population (EAS) obtained by the genome aggregation database gnomAD project. Here, we have shown the results for chromosome 6. As shown in Fig. 2a, the allele frequencies of SNVs in the two populations are highly correlated, as expected (Pearson correlation coefficient = 0.829). On the other hand, we could also observe some outliners. Table 2a describes several outliers falling in the regions marked as (i), (ii) and (iii) in Fig. 2a. Region (i) contained five outliners, two of which were located in low complexity regions as identified by RepeatMasker (4.0.0; http://www.repeatmasker.org/). A previous study^26^ suggested that the complexity of the genome sequence can affect the accuracy of short-read aligners, and thus it would be difficult to perform short-read sequencing analyses. Regions (ii) and (iii) contained approximately 20 SNVs in total, all of which were located around the HLA region (HLA-DQA1 gene and HLA-DQB1 gene). Again, these are known to be difficult regions for short-read analyses due to their high diversity^27,28^. In both cases, we think that most of the outliers resulted because of poor alignment of the short reads. Some of these outliers may be resolved upon reanalysis with next-generation long-read sequencers.

We also compared our reference panel with the RIKEN reference panel^29^ consisting of 2000 Japanese individuals, independent of our samples Fig. 2b. By comparing Fig. 2a and Fig. 2b, we can see a higher consistency between 3.5KJPNv2 and the RIKEN reference panel, although some outliners also exist in Fig. 2b. There are two main differences between our panel and the RIKEN panel. The first difference is at the filtration step for generating variant calls. We used VQSR for variant filtering after genotyping, while the RIKEN panel used several hard filters in addition to VQSR. We do not insist that VQSR is better than some combination of hard filtering, but we employed VQSR to reduce any bias introduced by pipeline differences. Another difference is that our panel was constructed for a general population, while the RIKEN panel was generated based on patient volunteers. We are not sure that this difference would have a large impact on the allele frequencies of most SNVs. However, we think it would be important to consider this difference when our panel is used for personalized healthcare. In other words, the allele frequencies of some rare variants can change due to this difference, which in turn can cause large differences in the ability to identify disease-causing variants and to evaluate genetic risks. Along with autosomes, we performed a comparison of X chromosome allele frequency determined by using two PARs and XTR, between gnomAD EAS and 3.5KJPNv2 Fig. 2c. In Fig. 2c, we can see a high correlation between two populations. We also manually investigated several outliers in Fig. 2c; however, we could not determine their causes. Manually investigated outliers are listed in Supplementary Table 2.

We also checked the distribution of Ts/Tv metrics for 3.5KJPNv2 and for the 1000 Genomes for each chromosome. In Fig. 2d, the horizontal axis represents the chromosomes, and the vertical axis shows the ratio of transitions and transversions (Ts/Tv ratio). Violin plots indicate the distributions of Ts/Tv ratios among individuals included in 3.5KJPNv2. The red dots represent Ts/Tv ratios for each chromosome compared against all samples in the East Asian genomes in the 1000 Genomes project, while the green dots represent comparison against the JPT samples (Japanese samples taken from Tokyo, Japan) included in the 1000 Genomes project. As a result, most of the Ts/Tv values, except for those obtained for chromosome 5, were highly similar to those obtained by the 1000 Genomes project, though slightly higher.

### Analysis of population structure

To observe the population structure in 3.5KJPNv2, we created a PCA plot for individuals included in 3.5KJPNv2 and the East Asian populations included in the 1000 Genomes Project. For the East Asian populations, we used the 1000 Genomes Project Phase 3 genotype data, available in the VCF format, for the following populations: CHB (Han Chinese in Beijing, China), JPT (Japanese in Tokyo, Japan), CHS (Southern Han Chinese), CDX (Chinese Dai in Xishuangbanna, China), and KHV (Kinh in Ho Chi Minh City, Vietnam). We obtained a combined genotype dataset by converting the genotype dataset of 3.5KJPNv2 and the dataset of East Asian populations by PLINK. During the conversion, variants with MAF < 0.01 or HWE < 1.0e-5 were removed. The two resultant BED files were combined on commonly existing variants. For the combined dataset, we removed variants with MAF < 0.05, HWE < 0.05, or those with a missing rate > 0.01. PCA was applied after LD pruning for the remaining variants with PLINK by selecting the “--indep-pairwise 200 4 0.1” option. We used the same PLINK parameters as Nagasaki et al^5^.

**Fig. 3:**
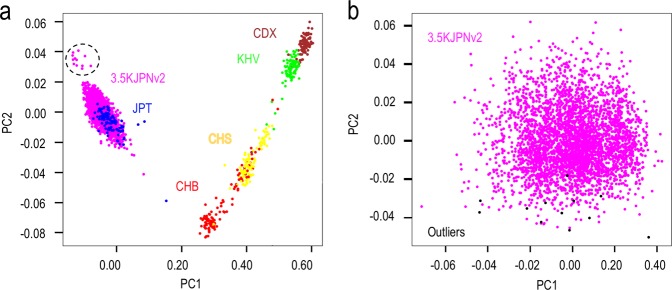
Population structure of 3.5KJPNv2. **a** PCA plot of 3.5KJPNv2 with the East Asian populations CHB, CHS, KHV, and CDX from 1KGP. We observed 12 outliers in the ToMMo samples. **b** PCA plot of 3.5KJPNv2 only. Black dots correspond to the outliers found in **a**

In the PCA plot Fig. 3a, the East Asian populations CHB, CHS, KHV, and CDX were aligned according to their geographical relationship. The 3.5KJPNv2 individuals and the JPT population overlapped with each other and formed a separate cluster from the CHB, CHS, KHV, and CDX populations. Although another small separate cluster of 12 individuals was found in the bottom left part of the larger cluster of 3.5KJPNv2 individuals, we did not observe high pairwise IBD values among these individuals. These were at most 0.0223 IBD according to estimation using the PLINK “–genome” option. In addition, these 12 individuals did not form a cluster in the PCA plot of only 3.5KJPNv2 individuals Fig. 3b.

### Availability of allele frequency panel with web interface

3.5KJPNv2 is distributed from jMorp (Japanese Multi Omics Reference Panel) with a web interface. jMorp was originally published as a database of metabolites and proteins in plasma obtained from volunteers in ToMMo, which was already described by Tadaka et al^30^. From jMorp release 201806 (Jun 2018, https://jmorp.megabank.tohoku.ac.jp/201806/), genomic variant data have been added, and the latest version 201902 (Feb 2019, https://jmorp.megabank.tohoku.ac.jp/201902/) is where allele frequencies of all the genomic variants can be examined through the web interface. Adding genomic variant information further enhances multilayer omics analysis. Details of the web usage are described in the tutorial section of the web page at https://jmorp.megabank.tohoku.ac.jp/201902/help/tutorial.

3.5KJPNv2 is available at the jMorp website with web interface, and the raw data in VCF (Variant Call Format) format was also registered at the NBDC Human Database (https://humandbs.biosciencedbc.jp/en/) with accession code hum0015.v3 by the National Bioscience Database Center (NBDC) of the Japan Science and Technology Agency (JST) to ensure accessibility, preservation and stability of the 3.5KJPNv2 datasets.

Individual’s sequence data and genotyping results from which allele frequency dataset is constructed and validated are available upon request after approval of the Ethical Committee and the Materials and Information Distribution Review Committee of Tohoku Medical Megabank Organization.

## Supplementary information

Supplemental Material

## Data Availability

In-house codes for this analysis are available on GitHub: https://github.com/gpc-gr/panel3552-scripts. Third-party software employed in this workflow is described in the Methods section.
