# Supplementary material for: 3.5KJPNv2: an allele frequency panel of 3552 Japanese individuals including the X chromosome
Source: Hum Genome Var. 2019 Jun 18;6:28. doi: 10.1038/s41439-019-0059-5 (PMC6581902; doi:10.1038/s41439-019-0059-5)

3.5KJPNv2, An allele frequency panel of 3,552 Japanese Individuals including X chromosome

Shu Tadaka[1,2,+], Fumiki Katsuoka[1,2,+], Masao Ueki[1,3], Kaname Kojima[1,2,4], Satoshi Makino[1,2], Sakae Saito[1,2], Akihito Otsuki[1,2], Chinatsu Gocho[1], Mika Sakurai-Yageta[1,2], Inaho Danjoh[1,2], Ikuko N. Motoike[1,2,4], Yumi Yamaguchi-Kabata[1,2], Matsuyuki Shirota[1,2,4], Seizo Koshiba[1,2], Masao Nagasaki[1,2,4], Naoko Minegishi[1,2,9], Atsushi Hozawa[1,2], Shinichi Kuriyama[1,2,5], Atsushi Shimizu[6], Jun Yasuda[1,7], Nobuo Fuse[1,2], the Tohoku Medical Megabank Project Study Group[10], Gen Tamiya[1,3], Masayuki Yamamoto[1,2,9], Kengo Kinoshita[1,4,8,9,*]

1. Tohoku Medical Megabank Organization, Tohoku University, Japan
2. Graduate School of Medicine, Tohoku University, Japan
3. Statistical Genetics Team, RIKEN Center for Advanced Intelligence Project, Japan
4. Graduate School of Information Sciences, Tohoku University, Japan
5. International Research Institute of Disaster Science, Tohoku University, Japan
6. Iwate Tohoku Medical Megabank Organization, Iwate Medical University, Japan
7. Miyagi Cancer Center, Miyagi Hospital Organization, Japan
8. Institute of Development, Aging and Cancer, Tohoku University, Japan
9. Advanced Research Center for Innovations in Next-Generation Medicine, Tohoku University, Japan
10. https://www.megabank.tohoku.ac.jp/english/a180601/

+ These authors contributed equally

* corresponding author: Kengo Kinoshita, 2-1, Seiryo-Machi, Aoba-ku, Sendai 980-8573, Tel: +81-22-274-6040, email: kengo@ecei.tohoku.ac.jp

Supplementary tables

Supplementary Table 1. Summary of samples included in 3.5KJPNv2

(a) The number of participants included in each project along with their presumed origin based on the birth place of their mother.

<table>
<tr><th>Participant group</th><th colspan="2">Subtotal</th></tr>
<tr><td>TMM Project</td><td colspan="2"></td></tr>
<tr><td>Miyagi or Iwate prefectures</td><td>1,507</td><td rowspan="3">3,342</td></tr>
<tr><td>Other than Miyagi and Iwate prefectures</td><td>491</td></tr>
<tr><td>Origin information is not available</td><td>894</td></tr>
<tr><td>Nagahama Study</td><td colspan="2">29</td></tr>
<tr><td>Individuals recruited in Nagasaki Medical Center</td><td colspan="2">181</td></tr>
<tr><td>Total</td><td colspan="2">3,552</td></tr>
</table>

(b) Participants' gender and sex. Age information for 14 participants was missing.

Age ranges	Male	Female	Total
**29 and younger**	66	173	239
**30-39**	184	329	513
**40-49**	124	243	367
**50-59**	219	402	621
**60-69**	629	653	1,280
**70-79**	312	194	506
**80 and older**	7	5	12

Supplementary Table 2. Overview of outliers found in allele frequency comparison plots in Fig. 2(c). The “Position” column shows the chromosomal position of a variant, the “Ref/Alt” column gives the reference allele and the alternative allele, the “3.5KJPNv2” column gives the allele frequency observed in 3.5KJPNv2, the “gnomAD EAS” column gives the allele frequency observed in gnomAD EAS.

	**Position**	**Ref/Alt**	**3.5KJPNv2**	**gnomAD EAS**	**Note**
**Fig. 2(c)**	18231907	C/T	0.3793	0.1164	rs45563640, BEND2 synomymous
	15792527	T/A	0.5129	0.2445	rs4364812, CA5B splice region
	39922359	A/G	0.7161	0.2774	rs3810694, BCOR intron
	135961587	G/T	0.7795	0.5047	rs2011584, RBMX missense
	69504078	T/G	0.8687	0.5768	rs12556489, RAB41 intron
	153048657	A/C	0.8273	0.5273	rs2314343, SRPK3 intron
	228940	A/G	0.2263	0.5000	rs62580112, GTPBP6 intron
	100749127	A/G	0.3637	0.6647	rs5951336, ARMCX4 missense
	28513629	G/C	0.8209	1.0000	rs5951336

Supplementary Figures

Supplementary Figure 1. Histogram of concordance ratio between whole genome sequencing results and SNP array genotypes.

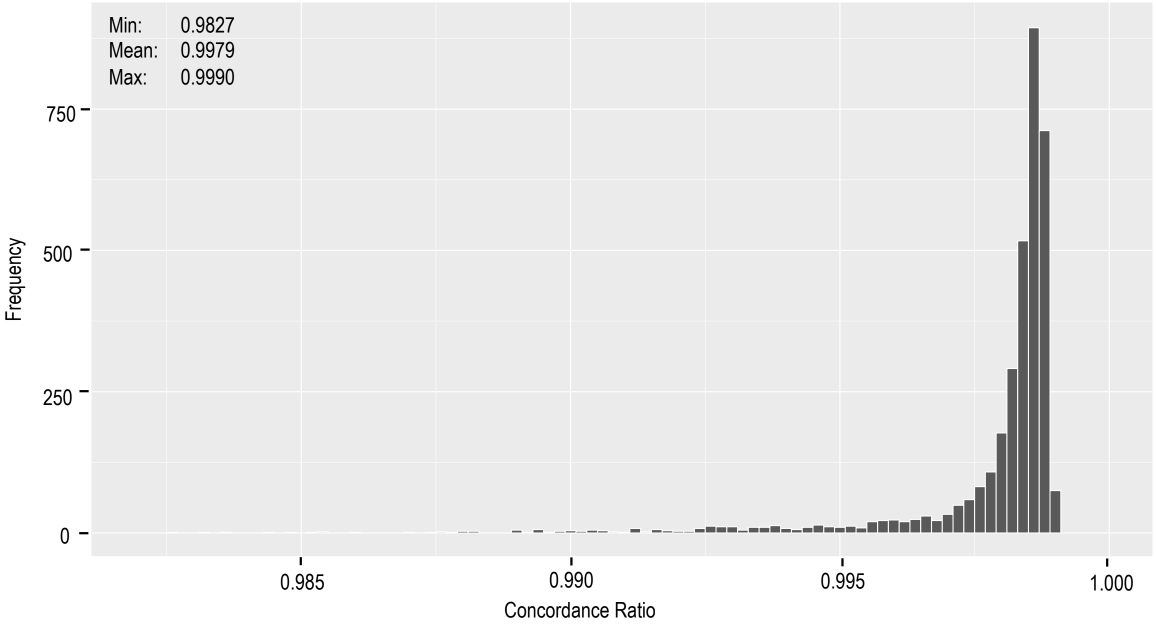

Supplement: Supplementary file 1 — Supplemental Material [file 41439_2019_59_MOESM1_ESM.pdf]
